# Establishment of topographic circuit zones in the cerebellum of scrambler mutant mice

**DOI:** 10.3389/fncir.2013.00122

**Published:** 2013-07-22

**Authors:** Stacey L. Reeber, Courtney A. Loeschel, Amanda Franklin, Roy V. Sillitoe

**Affiliations:** Department of Pathology and Immunology, Department of Neuroscience, Baylor College of Medicine, Jan and Dan Duncan Neurological Research Institute of Texas Children's HospitalHouston, TX, USA

**Keywords:** positional map, circuitry, topography, disabled1, connectivity, cerebellum

## Abstract

The cerebellum is organized into zonal circuits that are thought to regulate ongoing motor behavior. Recent studies suggest that neuronal birthdates, gene expression patterning, and apoptosis control zone formation. Importantly, developing Purkinje cell zones are thought to provide the framework upon which afferent circuitry is organized. Yet, it is not clear whether altering the final placement of Purkinje cells affects the assembly of circuits into topographic zones. To gain insight into this problem, we examined zonal connectivity in *scrambler* mice; spontaneous mutants that have severe Purkinje cell ectopia due to the loss of reelin-disabled1 signaling. We used immunohistochemistry and neural tracing to determine whether displacement of Purkinje cell zones into ectopic positions triggers defects in zonal connectivity within sensory-motor circuits. Despite the abnormal placement of more than 95% of Purkinje cells in *scrambler* mice, the complementary relationship between molecularly distinct Purkinje cell zones is maintained, and consequently, afferents are targeted into topographic circuits. These data suggest that although loss of disabled1 distorts the Purkinje cell map, its absence does not obstruct the formation of zonal circuits. These findings support the hypothesis that Purkinje cell zones play an essential role in establishing afferent topography.

## Introduction

The cerebellum is organized into an array of sagittal zones that control sensory-motor behavior (Seoane et al., 2005; Pijpers et al., 2008; Apps and Hawkes, 2009; Horn et al., 2010; Cerminara and Apps, 2011). Zones are best revealed by the expression patterns of genes and proteins in Purkinje cells (Apps and Hawkes, 2009; White et al., 2012; White and Sillitoe, 2013). For example, the Purkinje cell antigen zebrinII/aldolase C (Brochu et al., 1990; Ahn et al., 1994) is expressed in a striking array of well-defined zones (Sillitoe and Hawkes, 2002). ZebrinII zones are integrated into a broader map where they have an intricate relationship to the expression of several other Purkinje cell proteins. In some cases, zebrinII zones are complementary to the expression pattern of proteins such as phospholipase C β4 (PLCβ4; Armstrong and Hawkes, 2000; Sarna et al., 2006), while in other cases they are co-expressed with proteins such as phospholipase C β3 (PLCβ3; Armstrong and Hawkes, 2000; Sarna et al., 2006). Zones are defined not only by Purkinje cell expression but also by the organization of their axonal inputs. Each zone is innervated by a specific subset of climbing fiber afferents that directly contact Purkinje cells (Apps and Hawkes, 2009; White and Sillitoe, 2013).

Although we are beginning to understand how Purkinje cell zones form, our understanding of how the cerebellar cortical circuit is assembled into zonal connectivity patterns remains unclear. The leading hypothesis is that Purkinje cell patterning may restrict cerebellar interneurons and afferent projections to topographic zones (Sillitoe and Joyner, 2007; Apps and Hawkes, 2009). If Purkinje cell zones do provide the platform for zone assembly, then incoming afferents should be sensitive to the dispersal of Purkinje cells into their final locations. Here, we test the hypothesis that altering Purkinje cell placement will disrupt the assembly of circuits into distinct sagittal zones. We test this hypothesis using mice with defective reelin signaling because this molecular pathway has an established role in controlling the dispersal of embryonic Purkinje cells into a perfect monolayer in mature mice (Howell et al., 1997; Larouche and Hawkes, 2006; Miyata et al., 2010).

The spontaneous mutant *scrambler* contains an autosomal recessive mutation in the gene that encodes disabled1 (dab1), an adaptor protein that is essential for reelin signaling (Goldowitz et al., 1997; Howell et al., 1997; Sheldon et al., 1997; Rice et al., 1998). In the cerebellum, Purkinje cells selectively express disabled1 (Gallagher et al., 1998; Rice et al., 1998). Loss of disabled1 in mice disrupts cerebellar morphogenesis and causes severe ataxia (Sweet et al., 1996). The *scrambler* cerebellum is small and the lobules never develop because the size of the granule cell population is severely diminished by ~80% and more than 95% of Purkinje cells fail to complete their migration into a monolayer (Goldowitz et al., 1997). As a result, most Purkinje cells are located in ectopic masses within the central core of the cerebellum (Goldowitz et al., 1997). Although climbing and mossy fiber afferents terminate within ectopic Purkinje cell masses in *reeler* mutant mice (Blatt and Eisenman, 1988; Vig et al., 2005), it is not clear whether zonally organized afferents are targeted into molecularly distinct Purkinje cell zones. In this study, we exploit the *scrambler* mouse as a model for disrupting cerebellar patterning to ask whether zonal circuits are established despite the dramatic displacement of Purkinje cells into ectopic zones that are located within the central core of the cerebellum.

## Materials and methods

### Mice

All animal studies were carried out under an approved IACUC animal protocol according to the institutional guidelines at Albert Einstein College of Medicine and Baylor College of Medicine. Female and male *neuropeptide Y* (*Npy-Gfp*) transgenic mice (Pinto et al., 2004; Nishiyama et al., 2007) and female *scrambler* mice, which lack the *disabled1* gene (*Dab1^scm/scm^*; Sweet et al., 1996), were obtained from The Jackson Laboratory, (Bar Harbor, ME) and maintained in our colony on a C57BL/6J background. Here, we refer to the *disabled1* mutants as *scrambler*. The *scrambler* and *Npy-Gfp* strains were intercrossed to generate *scrambler:Npy-Gfp* double transgenic mice in order to genetically mark climbing fibers in *scrambler* mutants (*n* = 12 homozygous *scrambler:Npy-Gfp* mutants). Mice carrying the *Npy-Gfp* allele were identified by genotyping using a standard polymerase chain reaction with primers designed to detect *Gfp* (GFP 5′ sense: CTGGTCGAGCTGGACGGCGACG, GFP 3′antisense: CACGAACTCCAGCAGGACCATG and the expected band size is ~ 600 bp). *scrambler:Npy-Gfp* mice were genotyped for *Gfp*, phenotyped based on the severe postnatal ataxia observed in reelin pathway mutants (Goldowitz et al., 1997), and analyzed at 1-2 months of age. For anterograde tracing in developing animals (see below for the details of the procedure), whole litters were collected at postnatal day (*P*) 4/5 from *scrambler* heterozygote X heterozygote crosses, and each pup in the litter injected with tracer. Homozygous mutant pups were identified upon dissection, based on their small cerebella and well-understood lobule dysmorphology compared to littermate controls (Gallagher et al., 1998). Noon on the day a vaginal plug was detected was considered embryonic day (*E*) 0.5. The day of birth was designated as postnatal day 0 (P0). Adults were designated as P28 or older.

### Immunohistochemistry

Mice were anesthetized with avertin. Once all reflexes were abolished (e.g., lack of blink and corneal reflexes), the blood was flushed through the heart by perfusing with 0.1 M phosphate buffered saline (PBS; pH7.2). The tissue was then fixed by perfusing with 4% paraformaldehyde (4% PFA) diluted in PBS. The brains were then postfixed for 24–48 h in 4% PFA and then cryoprotected in buffered sucrose solutions (15 and 30% diluted in PBS). Serial 40 μm thick coronal sections were cut on a cryostat and collected as free-floating sections in PBS. Immunohistochemistry was carried out as described previously (Reeber et al., 2011). Briefly, tissue sections were washed thoroughly, blocked with 10% normal goat serum (NGS; Sigma, St. Louis MO, USA) for 1 h at room temperature and then incubated in 0.1 M PBS containing 10% NGS, 0.1% Tween-20 and the primary antibodies (see below) for 16–18 h at room temperature. The tissue sections were then washed three times in PBS and incubated in secondary antibodies (see below) for 2 h at room temperature. The tissue was rinsed again and immunoreactivity revealed as described below.

Monoclonal anti-zebrinII (Brochu et al., 1990) was used directly from spent hybridoma culture medium at a concentration of 1:250 (gift from Dr. Richard Hawkes, University of Calgary). Rabbit polyclonal anti-PLCβ4 (1:250) was purchased from Chemicon (Temecula, CA, USA), and revealed an identical pattern of Purkinje cell zones to what has previously been described (Sarna et al., 2006). Mouse monoclonal anti-Vesicular Glutamate Transporter 2 (VGLUT2; 1:500; Cat. # MAB5504) was purchased from Chemicon (Millipore; Billerica, MA) and was used to visualize climbing and mossy fiber terminals (Hisano et al., 2002; Gebre et al., 2012; Reeber and Sillitoe, 2011). Rabbit anti-GFP (1:1000) was purchased from Molecular Probes (Invitrogen; Carlsbad, CA). Chicken anti-GFP (1:2000) was purchased from Abcam (Cambridge, MA).

We tested three different anti-Npy antibodies on mouse sections at various concentrations (1:100 1:250 1:500, 1:1000, and 1:2000). Anti-Npy (Cat. # NAY-8060-V) was purchased from Peptides International (Louisville, KY) and has been shown to label zones in the rat cerebellum (Ueyama et al., 1994). The second Npy antibody tested was a rabbit polyclonal (Cat. # sc-28943) purchased from Santa Cruz (Dallas, TX). The third antibody tested was a rabbit polyclonal (NB600-1094) that was purchased from Novus Biologicals (Littleton, CO).

We visualized immunoreactive complexes either using diaminobenzidine (DAB; 0.5 mg/ml; Sigma, St. Louis, MO, USA) or fluorescence. For the DAB reaction we used horseradish peroxidase (HRP) conjugated goat anti-rabbit secondary antibodies (diluted 1:200 in PBS; DAKO, Carpinteria, CA, USA) to bind the primary antibodies. Staining for fluorescent immunohistochemistry was carried out using Alexa 488- and 555-conjugated immunoglobulins (Molecular Probes Inc., Eugene, OR, USA), both diluted to 1:1500. Tissues sections were coverslipped using either Entellan mounting media (DAB; Electron Microscopy Sciences, Hatfield, PA) or FLUORO-GEL with Tris buffer (Electron Microscopy Sciences, Hatfield, PA). We tested the specificity of the secondary antibodies by processing the tissue sections in the absence of primary antibodies. No signal was detected in such control experiments indicating that the staining we observed was not due to non-specific signals from the Alexa or HRP conjugated antibodies (data not shown).

### Anterograde tracing

Anterograde tracing was performed according to previous protocols (Sillitoe et al., 2010; Reeber et al., 2011). Approximately 250 nl of a 2% solution of WGA (wheat germ agglutinin) conjugated to Alexa Fluor 555 or 488 (Cat. #W32464 and Cat. #W11261, Invitrogen, Carlsbad, CA) was pressure injected into the lower thoracic-upper lumbar spinal cord of P4/P5 (*n* = 5 for each genotype) or adult mice (*n* = 5 for each genotype). After a 24 h (pups) or a 48 h (adults) survival period the mice were anesthetized as described above and then perfused with 4% PFA (described above). WGA-Alexa traced neurons are visible immediately upon cellular uptake and thus no additional tissue staining is required for labeling. Therefore, after the perfusion, the WGA-Alexa traced tissue was either cut and mounted for imaging or further processed for immunohistochemistry in order to examine the relationship between afferent projections and Purkinje cell zones. After tracing, the spinal cord was also cut in order to examine the size of the injection spot (local injections that span only one vertebral segment are ideal for pattern analysis) and to ensure that only limited tissue damage was caused by the injection (Reeber et al., 2011).

### Statistical analysis

In wild type mice, WGA-Alexa Fluor 555 accumulates as punctate deposits in mossy fiber terminals, which highlights the structure of the large terminal rosettes (arrowheads in Figures 3A,B; Reeber and Sillitoe, 2011; Reeber et al., 2011). The number of WGA-Alexa Fluor 555 labeled mossy fiber terminals were computed based on an arbitrarily determined boundary that fits within the limits of a single Purkinje cell zone on 40 μm cut sections (all counts were restricted to within 200 μm in the anterior-posterior axis of the cerebellum). At least 3 wild type and 3 *scrambler* tissue sections were used for the analysis (*n* = 5 animals/genotype). The number of WGA-Alexa labeled mossy fiber terminals, which were identified as large terminal rosettes, were counted on each tissue section. The sum of the number of mossy fiber terminals for each region was computed and the mean for each region was used to calculate the standard error of the mean, SEM. The p value was acquired using an unpaired *t*-test (GraphPad Prism) to compare the differences in the number of terminals between wild type and *scrambler* mutants.

For climbing fiber analysis, we acquired images from 4 to 5 sections through the anterior cerebellum of wild type and *scrambler* mutants (3 animals each) that were doubled stained for Gfp and PLCβ4, and then measured the distance of a medial-lateral region of cortex that was occupied by zones that were stained using each antibody. The regions that were heavily stained for Gfp revealed the most obvious climbing fiber zones. We computed a region of overlap between the two markers—the mean percentage overlap in a given region was used to calculate the SEM. The p value was acquired using an unpaired *t*-test (GraphPad Prism) to compare the difference in percent overlap between afferent zones and Purkinje cell zones in wild type versus *scrambler* mutants. *P* < 0.05 was considered to be significant.

### Microscopy and data analysis

Photomicrographs of tissue sections were captured using Leica DFC360 FX (fluorescence) and DFC 490 (DAB reacted tissue sections) cameras mounted on a Leica DM5500 microscope. Images of tissue sections were acquired and analyzed using Leica Application Suite and Leica Application Suite FX software packages supplemented with a deconvolution module. For deconvolution, stacks of 10–20 sections in the z-axis were collected every 0.2–0.5 μm. The z stack images were deconvolved (~10 iterations) and then analyzed as compressed projections or as individual slices. All raw data was imported into Adobe Photoshop CS4 and corrected for brightness and contrast only. Schematics were created in Adobe Illustrator CS4.

## Results

### Ectopic purkinje cells organize into molecularly distinct zones in scrambler mutants

**Figure 1:**
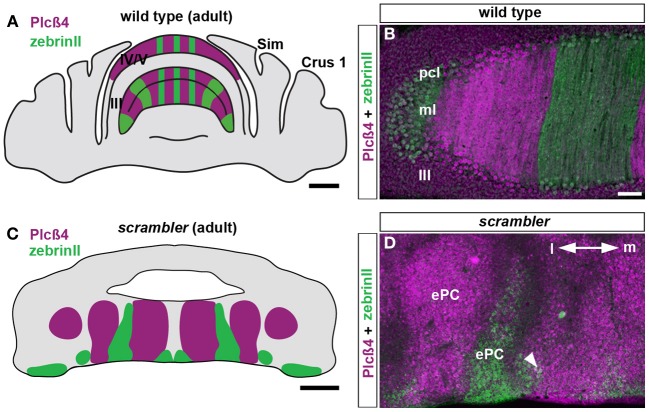
**ZebrinII and PLCβ4 expressing Purkinje cells are organized into discrete domains in adult *scrambler* mutants. (A,B)** The expression pattern of PLCβ4 is complementary to the expression pattern of zebrinII as observed on adult coronal tissue sections cut through the wild type mouse cerebellum. **(C,D)** In adult *scrambler* mutants, PLCβ4 and zebrinII zones are present in relatively normal configurations (i.e. complementarity between zones is maintained) but are organized into ectopic Purkinje clusters. The boundaries between zebrinII and PLCβ4 are poorly delineated and in some regions, we observed overlapping expression (arrowhead in **D**). Lobule numbers are indicated by Roman numerals. Abbreviations: pcl, Purkinje cell layer; ml, molecular layer; ePC, ectopic Purkinje cells; m, cerebellar medial; l, lateral. Scale bar in **A** = 1 mm; **B** = 100 μm (applies to **D**); **C** = 500 μm.

In *reeler* mice, zebrinII and p-path are expressed within alternating zones of ectopic Purkinje cells (Edwards et al., 1994). Similarly, removal of the reelin receptors, Vldlr and Apoer2, results in a large population of ectopic Purkinje cells that organize into zebrinII and PLCβ4 zones (Larouche et al., 2008). Removal of disabled1, which functions downstream of the Vldlr and Apoer2 receptors, also induces ectopic Purkinje cell clusters that express zebrinII. In addition, loss of diabled1 results in ectopic clusters of HSP25 expressing Purkinje cells (Armstrong et al., 2009). In this study we found that in *scrambler* mutants, zebrinII and PLCβ4 form a parasagittal zonal pattern, similar to, but distorted compared to the wild type map (Figures 1A–D; Gallagher et al., 1998). The boundaries between zebrinII and PLCβ4 zones are poorly delineated, and in some cases we observed domains with overlapping expression where Purkinje cells expressing zebrinII were extensively intermingled with those expressing PLCβ4 (arrowhead Figure 1D). The relationship between ectopic zones was replicated on either side of the cerebellar midline and the major zones were symmetrically distributed across the medial-lateral extent of the cerebellum (Figures 1C,D). Importantly, the overall zonal pattern in *scrambler* mutants was consistent between individuals (*n* = 8; Figures 1C,D). These data support the idea that the fundamental map of molecular zones is present in reelin signaling mutants, and despite the Purkinje cell ectopia the individual relationships between zones are maintained.

### Mossy fibers are targeted into zones of ectopic purkinje cells in scrambler mutants

During development, Purkinje cells are thought to establish zonal circuits by guiding the targeting of sensory projections (Apps and Hawkes, 2009). Accordingly, afferents in adults form a precise topographic map that respects the Purkinje cell zone boundaries (Gravel et al., 1987; Gravel and Hawkes, 1990; Akintunde and Eisenman, 1994; Zagrebelsky et al., 1996, 1997; Sawada et al., 2008; Armstrong et al., 2009; Pakan et al., 2010; Reeber and Sillitoe, 2011). We reasoned that if the Purkinje cell zonal map is fully represented in *scrambler* mutants, albeit in an almost entirely ectopic location, afferent fibers should be precisely targeted in ectopic zones if they depend on Purkinje cell organization for topographic targeting. To test the hypothesis that afferent connectivity respects Purkinje cell zonal boundaries despite the abnormal cytoarchitecture in *scrambler* mice, we analyzed the neuroanatomy of the spinocerebellar tract, which is a major sensory afferent pathway that projects to the cerebellum from all levels of the spinal cord (Arsenio Nunes and Sotelo, 1985; Sillitoe et al., 2010; Reeber and Sillitoe, 2011; Reeber et al., 2011).

**Figure 2:**
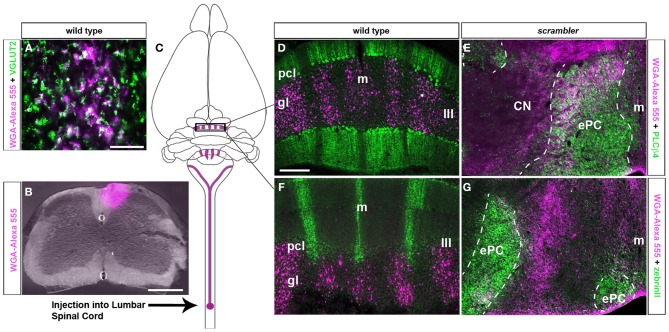
**Spinocerebellar mossy fiber targeting into longitudinal zones is conserved in *scrambler* mutant mice. (A)** VGLUT2 is a well-established pre-synaptic marker for mossy fiber terminals. Deconvolution microscopy demonstrates that VGLUT2 and WGA-Alexa 555 co-label a sub-population of spinocerebellar mossy fibers. **(B)** Image of an injection site after delivering WGA-Alexa 555 into the lower thoracic - upper lumbar region of the adult spinal cord. **(C)** The schematic illustrates the origin and termination of WGA-Alexa 555 labeled spinocerebellar neurons. **(D)** In wild type mice, WGA-Alexa 555 labeled mossy fibers terminals align with PLCβ4 immunoreactive Purkinje cells. **(E)** Similar to wild type mice, in *scrambler* mutant mice spinocerebellar fibers align with PLCβ4 immunoreactive Purkinje cell clusters. The dotted lines in panels E and G indicate the boundaries of PLCβ4 or zebrinII immunoreactive Purkinje cells. **(F)** In wild type mice, spinocerebellar fibers do not align with zebrinII immuoreactive Purkinje cells. **(G)** Ectopic spinocerebellar fibers in *scrambler* mutant mice do not innervate zebrinII immunoreactive Purkinje cell clusters. Abbreviations: ePC, ectopic Purkinje cells; m, midline; CN, cerebellar nuclei. Scale bar in **A = 50 μm;** in **B** = 500 μm; in **D** = 200 μm (applies to **D–G**).

The compartmental organization of spinocerebellar mossy fibers is a classic model for studying mossy fiber topography because its termination pattern clearly illustrates the degree to which cerebellar afferents are organized at the structural (Voogd et al., 1969; Arsenio Nunes and Sotelo, 1985; Yaginuma and Matsushita, 1989; Gravel and Hawkes, 1990; Vogel and Prittie, 1994; Reeber et al., 2011), developmental (Vig et al., 2005; Sillitoe et al., 2010; Reeber et al., 2011) and functional (Perciavalle et al., 1998; Valle et al., 2012) levels. Based upon these well-known characteristics of the pathway, we injected WGA-Alexa conjugated tracers into upper lumber-lower thoracic spinocerebellar neurons to examine the terminal field distribution of afferent fibers within ectopic Purkinje cells in *scrambler* mutants. Two days after injecting WGA-Alexa tracer into the lower thoracic-upper lumbar region of the spinal cord, mossy fiber axons and terminals were clearly revealed within the cerebellum (**Figures 2B,C**; *n* = 5 of each genotype; Reeber and Sillitoe, 2011; Reeber et al., 2011). We confirmed that WGA-Alexa 555 is expressed in mossy fiber terminals by co-labeling with VGLUT2, which is a pre-synaptic marker for mossy fiber terminals (**Figure 2A**; Hisano et al., 2002). In accordance with previous studies, spinocerebellar mossy fiber terminals in wild type mice project almost exclusively to the vermis and terminate selectively within lobules I–V and VIII/IX (Vogel and Prittie, 1994; Vig et al., 2005; Sillitoe et al., 2010; Reeber et al., 2011). Double labeling with PLCβ4 and zebrinII in WGA-Alexa 555 traced wild type mice revealed that spinocerebellar mossy fiber terminals align mainly with the PLCβ4 subset of Purkinje cells, and only minimally with zebrinII Purkinje cells (Figures 2D,F).

**Figure 3:**
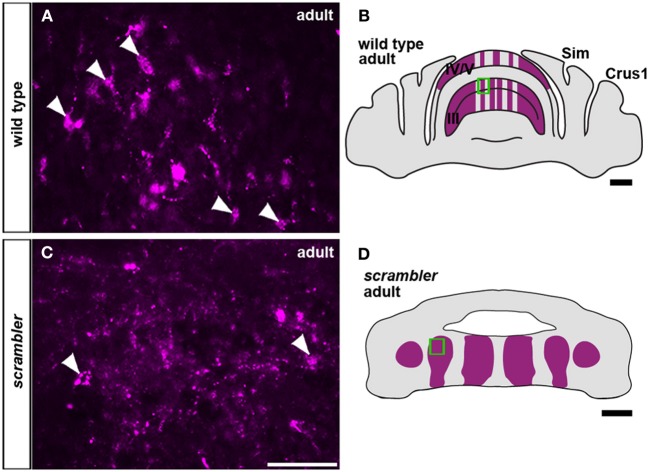
**Mossy fiber terminals within ectopic Purkinje cell clusters fail to form large grape-like glomeruli (rosettes). (A,C)** Anterogradely transported WGA-Alexa 555 accumulates as punctate deposits in mossy fiber axons and terminals. **(A,B)** High magnification image of WGA-Alexa 555 traced mossy fiber glomeruli in the granule cell layer of wild type adult mice (boxed area in **B**). The arrowheads are highlighting typical large terminal rosettes. **(C,D)** In *scrambler* adult mice, mossy fibers fail to differentiate into the complex “grape-like” glomerular structure. Arrowheads are pointing to relatively normal mossy fiber terminals within the ectopic cluster from the boxed region in **(D)**. Scale bar in **B** = 1 mm; **C** = 50 μm (applies to **A,C**); **D** = 500 μm.

Similar to wild type mice (Figures 2D,F), spinocerebellar mossy fiber terminals in *scrambler* mice heavily innervate zones of PLCβ4 immunoreactive Purkinje cells, although within ectopic positions (Figures 2E,G). Despite targeting appropriate Purkinje cell zones in *scrambler* mice, mossy fiber zone boundaries were not sharply delineated (Figures 2E,G) and the terminals within the ectopic clusters were morphologically abnormal. As we have previously reported in wild type mice, WGA-Alexa Fluor 555 accumulates as punctate deposits in mossy fiber terminals, which highlights the structure of the large terminal rosettes (arrowheads in Figures 3A,B; Reeber and Sillitoe, 2011; Reeber et al., 2011). In contrast, in adult *scrambler* mice only ~20% of the traced mossy fibers had the complex “grape-like” glomeruli structure that is typical of cerebellar mossy fibers (Figures 3C,D). Profile counts of large mossy fibers terminals revealed an average of 45 (±2.1) labeled rosettes within a specified region of cerebellar cortex in wild type mice, whereas *scrambler* mutants contained 9 (±1.0) labeled terminal rosettes (Figures 3C,D; *p* < 0.0001) within an equivalent sized region. We conclude that despite the lack of clear zonal boundaries and properly structured terminals, the topography of spinocerebellar afferent zones is not affected by the displacement of Purkinje cells that occurs after loss of disabled1 function in *scrambler* mutant mice.

### Early postnatal targeting of mossy fibers is not altered in scrambler mutants

**Figure 4:**
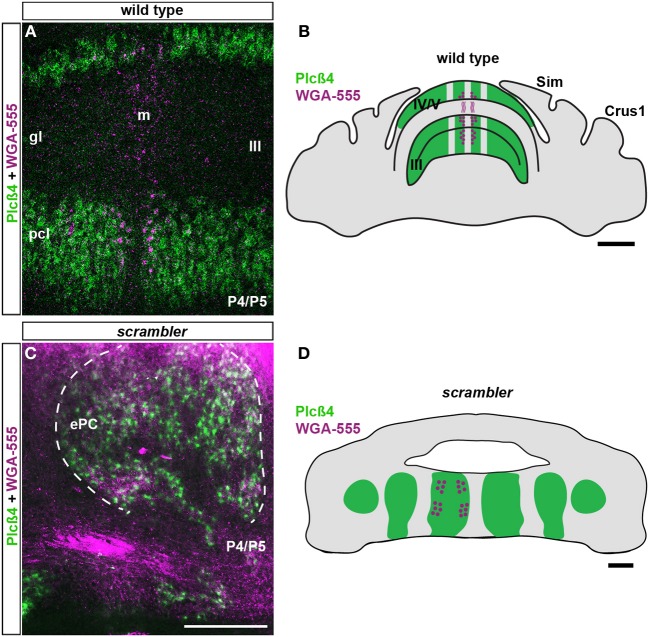
**Postnatal spinocerebellar afferents are topographically targeted in *scrambler* mutants. (A)**. In P4/5 wild type mice, WGA-Alexa 555 labeled mossy fiber terminals align with PLCβ4 immunoreactive Purkinje cells. **(C)** Similar to wild type mice, in P4/5 *scrambler* mutant mice spinocerebellar fibers align with PLCβ4 immunoreactive Purkinje cell clusters. The dotted line in panel C indicates the boundary of an ectopic PLCβ4 immunoreactive Purkinje cell cluster. **(B,D)** The schematic illustrates the termination of WGA-Alexa 555 labeled spinocerebellar mossy fibers in wild type **(B)** and *scrambler* **(D)** mice. Abbreviations: ePC, ectopic Purkinje cells; pcl, Purkinje cell layer; gl, granule cell layer. Scale bar in **B** = 750 μm; **C** = 200 μm (applies to **A, C**); **D** = 200 μm.

**Figure 5:**
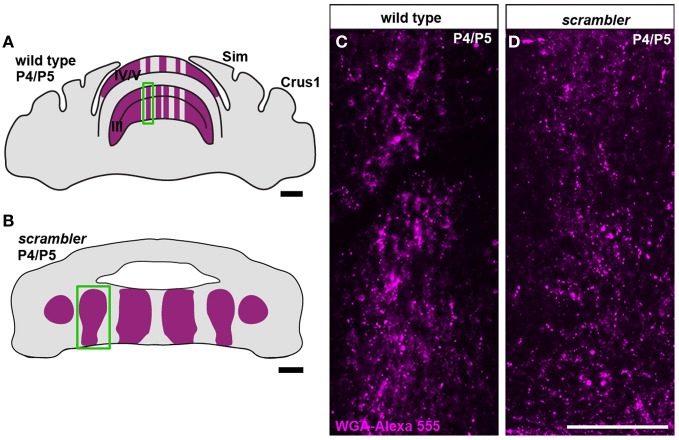
**Mossy fiber terminal structure is established in postnatal *scrambler* mice. (A,C)** Anterogradely transported WGA- Alexa 555 accumulates as punctate deposits in spinocerebellar mossy fiber axons and terminals in wild type P4/5 mice. **(B,D)** Punctate deposits were also observed in the terminals of P4/5 *scrambler* mutant mice. The images in panels **(C,D)** were taken from the boxed regions in panels **(A,B)**, respectively. Scale bar in **A** = 750 μm; **B** = 200 μm; **D** = 100 μm (applies to **C,D**).

Because mossy fiber zonal boundaries are poorly resolved in adult *scrambler* mutants, we wondered whether an altered developmental timetable could contribute to the formation of a distorted mossy fiber afferent map. To determine whether the initial patterning of mossy fibers into zones is altered in *scrambler* mutant mice, we used WGA-Alexa 555 to examine spinocerebellar tract development during the first week after birth. We previously determined that spinocerebellar mossy fibers resolve into a zonal pattern by ~P5 in mice (Sillitoe et al., 2010). Therefore, we injected mice with tracer at P4/P5 and then one day later we compared mossy fiber topography in wild type and *scrambler* mice (**Figure 4**; *n* = 5 of each genotype; Reeber and Sillitoe, 2011; Reeber et al., 2011). Consistent with our previous report, by P5 we observed rudimentary zones of spinocerebellar mossy fiber terminals within lobules I-V and VIII/IX in wild type mice. We also found that similar to the adult, P5 mossy fiber terminals align with PLCβ4 immunoreactive Purkinje cells (Figures 4A,B). Spinocerebellar mossy fiber terminals in *scrambler* mice also terminated in zones at P5. Strikingly, afferent terminals in *scrambler* were mainly targeted into ectopic PLCβ4 Purkinje cell clusters (Figures 4C,D). Note that in both wild type and *scrambler* mice scattered WGA-Alexa traced terminals can be seen in the PLCβ4 negative domains because afferent pruning is not complete at this stage of development. Unlike adult *scrambler* mice that have abnormal mossy fiber terminal structure, we observed that mossy fiber terminals in P4/5 *scrambler* mice have a comparable immature structure to wild type pups of the same age (Figures 5A–D). These data suggest that in *scrambler*, mossy fiber afferents resolve into zones at the right time, and they target the correct subsets of Purkinje cells. In addition, although mossy fibers in *scrambler* mutants may not differentiate properly, the gross anatomical features typical of granular layer presynaptic terminals do form. In summary, the spatial and temporal properties of spinocerebellar afferent zonation are not dependent on reelin-disabled1 signaling, although the structural features of clear-cut sharp zones with large terminal rosettes are altered when *disabled1* is deleted. Our data support the hypothesis that Purkinje cell patterning may be the major determinant of spinocerebellar afferent zone formation (Sotelo, 2004).

### Climbing fiber zones are revealed using Npy-Gfp transgene expression

**Figure 6:**
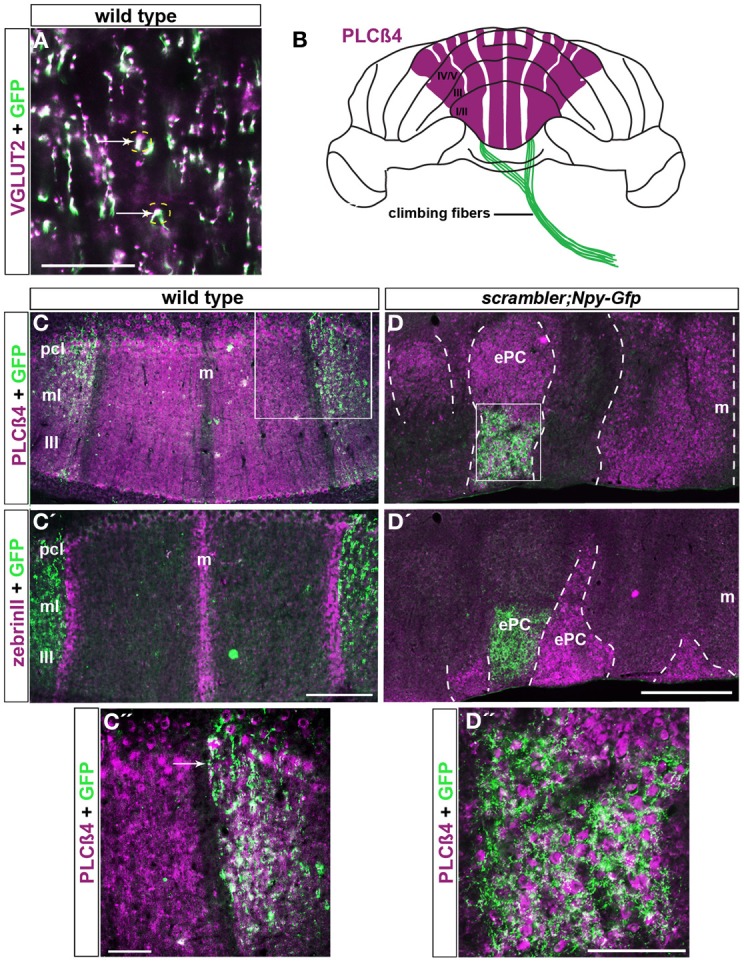
***Npy-Gfp* labeled climbing fiber topography is maintained in *scrambler* mutants. (A)**. VGLUT2 is a molecular marker known to label climbing fiber terminals. VGLUT2 and *Npy-Gfp* co-label a sub-population of climbing fibers (arrows). **(B)** Whole mount schematic illustrating the normal pattern of *Npy-Gfp* labeled climbing fibers innervating PLCβ4 immunoreactive Purkinje cell zones. **(C,C')** *Npy-Gfp* is expressed in two broad parasagittal zones that span ~500 μm on either side of the midline in adult wild type mice. **(C)** *Npy-Gfp* labeled climbing fiber zones overlap with PLCβ4 immunoreactive Purkinje cell zones. **(C')** *Npy-Gfp* labeled climbing fiber zones respect Purkinje cell stripe boundaries and do not invade the zebrinII immunoreactive Purkinje cell zones. **(D,D′)** *Npy-Gfp* is expressed in climbing fibers in *scrambler:Npy-Gfp* mutant mice. **(D)** Similar to wild type mice, in *scrambler* mice climbing fibers selectively innervate PLCβ4 immunoreactive Purkinje cell clusters. The dotted lines in panels **(D,D′)** indicate the boundaries of PLCβ4 and zebrinII immunoreactive Purkinje cells. **(D')** *Npy-Gfp* expressing climbing fibers do not target zebrinII labeled Purkinje cells in *scrambler* mutant mice. **(C″,D″)** High magnification images of boxed regions in **(C,D)** show that *Npy-Gfp* is expressed in climbing fiber zones that terminate upon PLCβ4 immunopositive Purkinje cells in both wild type and *scrambler* mutant mice. Scale bar in **A** = 60 μm; in **C'** = 250 μm (applies to **C–C**); in **C″**= 50 μm; in **D** = 500 μm (applies to **D–D′**); in **D″** = 100 μm.

Climbing fiber projections arise from cells in the inferior olivary complex of the brainstem and monoinnervate Purkinje cells. Although neuroanatomic approaches have been indispensible for studying olivo-cerebellar circuit architecture (Fujita and Sugihara, 2013), more precise methods are required for routinely examining climbing fiber circuit topography in normal and mutant mice. The surgical procedures for injecting tracers into the olive require skilled surgeries (Blatt and Eisenman, 1988) and routinely injecting the same sub-nuclei to reveal olivo-cerebellar topography is challenging (Sotelo et al., 1984). These challenges in studying olivo-cerebellar connectivity arise mainly because the ventral location of the inferior olive makes it hard to access during surgery. We thought that these problems could be overcome if reporter gene expression in an *Npy-Gfp* transgenic line (Nishiyama et al., 2007) labeled only a subset of climbing fibers (Figures 6A,C,C′). Npy protein is a known marker for climbing fibers zones in the rat cerebellum (Ueyama et al., 1994; Morara et al., 1997). Because Npy antibodies are unreliable in mouse, and the three we tested did not reveal positive staining (data not shown; see methods), we analyzed the *Npy-Gfp* transgenic line to test whether this transgene would label zones of climbing fibers (*n* = 6). We first confirmed the identity of genetically labeled afferents as climbing fibers by double labeling transgenic tissue sections with GFP and VGLUT2, an established marker for climbing fiber terminals (Figure 6A; Hisano et al., 2002). Climbing fiber axons were observed in the white matter and could be traced through the granular layer, and into the molecular layer where they terminated upon the proximal two thirds of Purkinje cell dendrites (Figure 6A).

Not all climbing fibers expressed the *Npy-Gfp* transgene. Labeled climbing fibers were most obvious in the anterior lobules of the cerebellum. In lobules I–V, the transgene is expressed in at least two zones of climbing fibers that are located on either side of the midline (Figures 6B–C″). The climbing fibers in both zones terminate selectively upon PLCβ4 immunopositive Purkinje cells and do not invade the adjacent zebrinII Purkinje cell zones (arrows in Figures 6C–C″). Thus, the afferent termination pattern of *Npy-Gfp* labeled climbing fibers follows the topographical relationship known to exist between climbing fibers and Purkinje cells (Apps and Hawkes, 2009). We propose that the *Npy-Gfp* transgenic mouse is a useful new tool for genetically labeling climbing fiber zones *in vivo*.

### Climbing fibers terminate within zones of ectopic purkinje cells in scrambler mutants

To further test the hypothesis that afferent fiber zones are correctly matched to Purkinje cell subtypes despite the abnormal cytoarchitecture in *scrambler* mice, we crossed the *Npy-Gfp* transgene onto the background of the *scrambler* allele to examine climbing fiber-Purkinje cell topography. We examined *scrambler*: *Npy-Gfp* mice, which are homozygous for the *scrambler* allele and hemizygous for the *Npy-Gfp* allele, for patterning defects (*n* =12). Despite the severe ectopia of Purkinje cells in *scrambler*, we found that *Npy-Gfp* labeled climbing fibers were nevertheless topographically aligned with specific Purkinje cell clusters (Figures 6D–D″). Double staining showed that in *scrambler* mutants, similar to wild type mice, the *Npy-Gfp* expressing climbing fibers selectively innervated PLCβ4 immunoreactive Purkinje cell clusters (Figures 6D,D″). Moreover, quantification of the overlap between a heavily expressing Gfp region located within a PLCβ4 zone revealed a 49% (±8%) overlap in an anterior zone of wild type mice and a 37% (±4%) overlap in a *scrambler* mutant zone (the difference between the genotypes was not significant at *p* = 0.2079). In adjacent regions, we observed close to 100% overlap between afferent and Purkinje cell zones (for both wild type and mutant; Figures 6C′,D″). Thus, although the percent overlap may be different depending on the region of cerebellum analyzed, the relationship is consistent between animals and equivalent in wild type versus mutants. Overall, these results reveal that the loss of reelin-disabled1 signaling, and the ectopic placement of Purkinje cells do not interfere with climbing fiber recognition of molecularly distinct Purkinje cells.

## Discussion

The cerebellum is organized into a complex array of topographic sagittal zones; zones are best defined by the molecular expression patterns in Purkinje cells (Apps and Hawkes, 2009). In this investigation, we sought to determine whether altering Purkinje cell patterning by genetically inducing severe ectopia affects the targeting of afferent fibers into specific zones. We used transgenic mice that express *Gfp* driven by a *neuropeptide Y* gene promoter and a novel WGA-Alexa tracing approach to label climbing fibers and mossy fibers in the developing and adult cerebellum. Using the spontaneous mutant *scrambler* as a model for Purkinje cell mis-patterning, we demonstrate that despite the severe ectopia of Purkinje cell zones, the topography of afferent zones is established and afferent-target specificity is maintained.

### Complex purkinje cell zonation is established in reelin signaling mutants

Cerebellar zones are clearly delineated by the patterned expression of several genes and proteins (Apps and Hawkes, 2009; White and Sillitoe, 2013). For example, zebrinII (adolase C; Ahn et al., 1994; Hawkes and Herrup, 1995) and PLCβ4 (Armstrong and Hawkes, 2000; Sarna et al., 2006) are expressed in complementary subsets of Purkinje cells. Mutations in the components of the reelin pathway cause severe morphogenetic abnormalities in mice, but the Purkinje cell zones that express 5'-nucleotidase, zebrinII, HSP25, and p-path are clearly represented within ectopic clusters (Caviness and Rakic, 1978; Goffinet, 1984; Eisenman, 1988; Edwards et al., 1994; Gallagher et al., 1998; Larouche et al., 2008; Armstrong et al., 2009). Thus, despite the severe Purkinje cell ectopia in *reeler*, *disabled1*, *Apoer2* and *Vldlr* mutants, Purkinje cell zonation is still established (Blatt and Eisenman, 1988; Vig et al., 2005; Larouche et al., 2008). Combined with our data, these studies suggest that the major molecular divisions of the cerebellar zonal map are represented in reelin signaling mutants, although within ectopic cellular clusters.

### Cerebellar afferents are topographically organized in scrambler mutant mice

The organization of parasagittal Purkinje cell zones is mirrored by the topography of mossy fiber (Gravel and Hawkes, 1990; Akintunde and Eisenman, 1994; Armstrong et al., 2009; Pakan et al., 2010) and climbing fiber (Gravel et al., 1987; Zagrebelsky et al., 1996, 1997; Sawada et al., 2008) terminal fields. The timing and precision of this relationship led to the hypothesis that Purkinje cell zones, which emerge in the embryo, determine the patterned organization of afferent projections during late embryogenesis and early postnatal development (Sillitoe and Joyner, 2007; Apps and Hawkes, 2009). In this study, we tested the role of Purkinje cell placement during zonal circuit formation by examining *scrambler* mice (Goldowitz et al., 1997; Howell et al., 1997; Sheldon et al., 1997; Rice et al., 1998). Despite the severe ectopia of Purkinje cells in *scrambler* mice, *Npy-Gfp* labeled climbing fibers and WGA-Alexa traced spinocerebellar mossy fibers terminate within severely ectopic positions although still in alignment with their normal targets, the PLCβ4 immunoreactive Purkinje cell clusters (Figures 2, 6). Accordingly, somatostatin immunoreactive mossy fibers correctly innervate HSP25-immunoreactive zones in *scrambler* and *weaver* mutant mice, which is another mouse strain with ectopic Purkinje cells (Armstrong et al., 2009). These data are consistent with observations from *reeler* mutants, in which climbing fiber, spinocerebellar, and vestibulocerebellar afferents were shown to terminate into relatively normal anterior-posterior locations (Vig et al., 2005).

We also observed that within the ectopic Purkinje cell clusters, mossy fiber terminals failed to differentiate into their typical complex “grape-like” structure (Figure 3). Surprisingly, despite this lack of apparent structural complexity at the synapse, *in vivo* recordings in *reeler* mutants demonstrated that afferents do make connections in the ectopic clusters and afferent information is transferred to their ectopic Purkinje cells with some level of fidelity (Mariani et al., 1977). Taken together, these data indicate that the major functional circuits of the cerebellum are established in reelin signaling mutants, and that Purkinje cell topography may influence several stages of cerebellar circuit formation.

### Afferent targeting is not altered in early postnatal scrambler mutant mice

Spinocerebellar mossy fibers enter the cerebellar anlage at approximately E13/14 in mouse (Grishkat and Eisenman, 1995) and at ~E12 in rat (Ashwell and Zhang, 1992, 1998). Mossy fiber topography is set up before most granule cells differentiate and migrate into the developing internal granular layer (Arsenio Nunes and Sotelo, 1985); during this time, mossy fibers directly contact clusters of immature Purkinje cells (Mason and Gregory, 1984; Grishkat and Eisenman, 1995; Sillitoe et al., 2010). It is postulated that mossy fibers disperse along with Purkinje cells as clusters transform into mature zones. During postnatal development, granule cells migrate past the Purkinje cells to form the internal granular layer, and then mossy fiber terminals translocate from Purkinje cells to granule cells (Sillitoe and Hawkes, 2013). This model is consistent with our observation that in *scrambler*, which lack ~80% of their granule cells, and in other agranular mutants, spinocerebellar mossy fiber topography is established despite the absence of a normal mossy fiber-granule cell-Purkinje cell circuit (Arsenio Nunes and Sotelo, 1985; Arsenio Nunes et al., 1988; Eisenman and Arlinghaus, 1991). We found that at P5, in *scrambler* mutants, mossy fibers are targeted into their “correct” circuits, although within ectopic Purkinje cell zones. Thus, as long as Purkinje cells can resolve into different chemical phenotypes, zonal circuit connectivity proceeds with limited disruption.

## Author contributions

Stacey L. Reeber, Courtney A. Loeschel, and Roy V. Sillitoe designed the experiments. Stacey L. Reeber, Amanda Franklin, and Roy V. Sillitoe performed the experiments. Stacey L. Reeber, Courtney A. Loeschel and Roy V. Sillitoe wrote the paper.

### Conflict of interest statement

The authors declare that the research was conducted in the absence of any commercial or financial relationships that could be construed as a potential conflict of interest.
